# Determinants of patients’ needs in asthma treatment: a cross-sectional study

**DOI:** 10.1038/npjpcrm.2016.44

**Published:** 2016-08-11

**Authors:** Adrian Loerbroks, Aziz Sheikh, Verena Leucht, Christian J Apfelbacher, Andrea Icks, Peter Angerer

**Affiliations:** 1 Institute of Occupational and Social Medicine, Centre for Health and Society, Faculty of Medicine, University of Düsseldorf, Düsseldorf, Germany; 2 Asthma UK Centre for Applied Research, Allergy and Respiratory Research Group, Centre of Medical Informatics, The University of Edinburgh, Medical School, Edinburgh, UK; 3 Division of General Internal Medicine and Primary Care, Brigham and Women's Hospital, Boston, MA, USA; 4 Department of Medicine, Harvard Medical School, Boston, MA, USA; 5 Medical Sociology, Department of Epidemiology and Preventive Medicine, University of Regensburg, Regensburg, Germany; 6 Division of Public Health and Primary Care, Brighton and Sussex Medical School, Falmer, UK; 7 Institute for Biometrics and Epidemiology, German Diabetes Center at the Heinrich-Heine University, Leibniz-Center for Diabetes Research, Düsseldorf, Germany; 8 Public Health Unit, Centre for Health and Society, Faculty of Medicine, University of Düsseldorf, Düsseldorf, Germany

## Abstract

Patients’ needs in asthma remain insufficiently understood and met. We therefore aimed to investigate the potential determinants of patients’ needs in asthma treatment. Our study was based on survey data on 189 adults with asthma. Needs were measured using the 13-item Needs in Asthma Treatment questionnaire, which yields a total score and subscale-specific scores (‘exacerbations’, ‘patient expertise’, ‘handling drugs’ and ‘drug effects’). We considered age, sex, education, years since diagnosis and anxiety/depression (measured by the Patient Health Questionnaire-4) as potential determinants. Associations were estimated by multivariable linear regression. Overall, we observed that younger age, poor mental health and a more recently established asthma diagnosis were independently associated with increased needs. Information on drug effects was an exception to this pattern as the need in that domain was solely determined by sex (being greater in men). In conclusion, our study provides novel evidence on patient characteristics that are associated with needs in asthma treatment. If confirmed by future studies, our observations may assist healthcare professionals to identify asthma patients with potentially elevated information, support and training needs and could contribute to the development of tailored interventions.

## Introduction

Asthma is a chronic respiratory condition characterised by airway inflammation and associated symptoms such as wheezing and shortness of breath.^1^ The burden of asthma is substantial in terms of its worldwide prevalence^2^ and cost.^3^ Epidemiological data have suggested that asthma remains poorly controlled in a significant proportion of patients.^4^ These observations have resulted in calls for a stronger emphasis on patient-focused care in asthma,^5^ which includes tailoring of treatment approaches to patient needs,^1^ such as their information, support and training needs. Addressing those needs (e.g., through shared decision making) may prove effective in improving asthma management and reducing morbidity.^6–8^

Despite their relevance, the needs of patients with asthma in relation to their condition remain insufficiently understood.^5,9–11^ In this context, insights into the patient characteristics associated with unmet needs are crucial in order to be able to identify patient subpopulations that may benefit most from addressing needs. Such information has the potential to inform the development of tailored patient-centred interventions. To our knowledge, factors that potentially determine patients’ needs in asthma treatment have not been examined to date. We therefore aimed to address this knowledge gap.

## Results

The sample was predominantly middle-aged (mean age=46.07 years with s.d.=16.04) and female (Table 1). About half of the participants had attained the highest educational level. Every fifth individual reported key symptoms of anxiety or depression and the Patient Health Questionnaire (PHQ)-4 mean score equalled 2.64 (s.d.=2.69). On average, respondents had been diagnosed with asthma 14.45 years (s.d.=13.29) prior to their survey participation, with about one-third having been diagnosed within the last 5 years. In the present study population, the prevalence of unmet needs was substantial: 11 out of 13 needs were considered unmet by at least 29% of the participants and the mean total need score equalled 0.36 (s.d.=0.27). The mean scores on the subscales drug effects, handling asthma drugs, exacerbations and patient expertise were 0.58 (s.d.=0.40), 0.17 (s.d.=0.28), 0.39 (s.d.=0.38) and 0.34 (s.d.=0.38), respectively.

As shown in Table 2, the mean total need score was 0.13 to 0.14 points higher in younger versus older participants, in those with symptoms of anxiety and/or depression (versus those free of these symptoms), and in those diagnosed with asthma within the last 5 years (versus >5 years). By contrast, education and sex were not associated with total needs in asthma treatment. In sub-scale-specific analyses, we found a comparable pattern of associations for information and training needs related to asthma exacerbations. Further, the need for consideration of the patient’s living conditions and expertise during diagnosis and treatment planning (i.e., ‘patient expertise’) was elevated in younger patients and in those with anxiety/depression, but not in those with a recent asthma diagnosis. The need for information and training on how to handle asthma drugs was more pronounced in younger participants and in those with a recently established diagnosis. The pattern of determinants for the need for information on asthma drug effects was distinct from the other domains: such information needs were solely determined by sex; that is, women expressed less need of such information compared with men.

## Discussion

### Main findings

We found that younger asthma patients, those with poor mental health, and patients who have received their diagnosis more recently, reported higher information, support and training needs. The single exception to this pattern was the need for information on drug effects, which was solely determined by sex.

### Interpretation of findings in relation to previously published work

Our observations related to the role of patients’ age, mental health status and how recently the asthma diagnosis had been made are in line with earlier findings, as suggested by a recent review of needs among patients with chronic diseases in German healthcare.^12^ Notably, that review did not include studies on asthma and was limited to information needs. Our findings thus expand that prior evidence to asthma and beyond information needs as the Needs in Asthma Treatment (NEAT) questionnaire covers a broader spectrum of needs. The increased needs in younger versus older patients may be explained by younger patients’ preferences for shared decision making^13^ and thus possibly represent a cohort effect. Our study suggests that symptoms of depression/anxiety are mainly linked to the need for consideration of the patient’s expertise and the need for information and training that assist in coping with exacerbations. In particular, the latter finding seems plausible given that exacerbations are a fear-laden topic among asthma patients.^11,14^ Further, prior qualitative work has highlighted that asthma patients consider asthma-related needs (e.g., provision of basic information) particularly salient around the time point when their diagnosis is being established.^9,11^ Our study confirms this hypothesis and suggests that novice asthma patients are in particular need of information and skills training related to coping with exacerbations and the correct handling of drugs. By contrast, novice patients do not seem to express an elevated need for consideration of their expertise, as suggested by our study: such expertise is likely limited at the point in time when the diagnosis is established and/or any such needs may be overshadowed by feelings of insecurity (e.g., emotional coping with the diagnosis and fear about the future).^11^ We observed that the need for information on drug effects (e.g., mechanisms and side effects) is more pronounced in men than in women. This observation should be interpreted with caution though: men were under-represented in our study and we cannot rule out that men participated more selectively than women, which may have contributed to that specific finding. Consequently, both sexes should still be considered in need of information on drug effects unless our finding is corroborated by multiple independent studies. Finally, we need to add that it has been suggested that a poor health status is associated with more pronounced information needs in patients with chronic disease.^12^ We have previously made this observation for asthma when we provided initial evidence of the validity of the NEAT questionnaire. In that prior work, we documented that all domains of unmet needs covered by the NEAT questionnaire were associated with poorer asthma control and poorer asthma-specific quality of life.^11^ The observations from the present report provide further evidence of the validity of the NEAT questionnaire.

### Strengths and limitations of this study

The limitations of our study deserve mentioning. First, we defined asthma based on participants’ reports of a physician diagnosis. Access to participants’ medical records or collection of additional clinical and physiological data would be desirable to enhance our asthma case definition. Notably though, self-reports of physician-diagnosed asthma are widely used asthma indicators in epidemiological research^4,15,16^ and have been found to be highly reliable^17^ and to show good agreement with the data from medical records.^18^ Further, the restriction of our sample to those not reporting chronic obstructive pulmonary disease (COPD) has likely limited the potential for diagnostic confusing of asthma and COPD.^19^ Second, our study is cross-sectional and as such care needs to be taken in making causal inferences. Third, our study sample was predominantly female and had high educational levels. We are unable to rule out that ‘hard-to-reach’ patients were not included or under-represented, which may limit our findings’ generalisability. Finally, it needs to be noted that we were unable to establish psychiatric diagnoses of depression or anxiety disorders in the current study. Our mental health assessment was limited to a short instrument (the PHQ-4^20^), which serves the purpose of merely screening for key symptoms of those conditions.

### Implications for future research, policy and practice

If confirmed by future studies, our observations may assist healthcare professionals to identify asthma patients with potentially elevated needs based, for instance, on their age, sex and the period of time since they received their asthma diagnosis. In addition, the insights from our study may help to inform the development of tailored interventions that aim to address the information, support and training needs of asthma patients. Additional research is, however, needed as we cannot rule out the possibility that there are more proximal determinants of asthma needs, which may be more suitable to tailor interventions. Such alternative determinants may include psychosocial factors, including asthma-related health literacy, knowledge, beliefs and self-efficacy^21–24^ (e.g., patients’ level of self-efficacy to proactively make their needs a subject of discussion with their physician).

### Conclusions

In summary, our study provides novel evidence on patient characteristics that determine patient needs in asthma.

## Materials and Methods

We used the data from a survey of 362 adults who reported to have ever been diagnosed with asthma by a physician.^11^ Participants were recruited through various pathways (e.g., physicians, pharmacies and patient organisations). To improve the sensitivity of our asthma case definition, the present analyses were restricted to participants who reported that they have never been diagnosed with COPD (*n*=189). Our study received approval from the Institutional Review Board of the Medical Faculty of the University of Düsseldorf. Informed consent was obtained from all participants. Our study adhered to the Helsinki Declaration on ethical principles for medical research involving human subjects.

Needs were measured by the NEAT questionnaire.^11^ The NEAT questionnaire was developed in a multi-stage process involving repeated focus groups with patients and cognitive interviews. Psychometric analyses suggested a 13-item instrument with the following four subscales:

- Drug effects: three questions on information needs related to how asthma drugs operate physiologically as well as the major potential side effects and pharmacological interactions with other drugs.
- Handling asthma drugs: three items assessing information and training needs related to the actual administration of asthma drugs (e.g., dosing and the need for training of the correct inhaler technique).
- Exacerbations: three items addressing needs related to how to respond to asthma attacks, specifically information needs and training needs (i.e., breathing techniques) among patients, as well as patients’ perception of information needs among their family members/friends.
- Patient expertise: four questions covering patients’ wishes that their physician considers their asthma knowledge and/or individual life circumstances to a larger extent and set aside more time in case patients have special requests.

All items are presented as questions (e.g., ‘would you like more information on…’) with the response options ‘Yes, I would like this’, ‘This need has already been met’ and ‘No, I do not need this’. For the current study, we coded responses as 1=yes versus 0=no and calculated mean scale-specific scores and a total score. The potential score range on each subscale and the total scale may thus vary from 0.0 to 1.0, with higher scores indicating higher need. The NEAT subscales and the total score showed good internal consistency, and increasing levels of unmet needs were associated with poorer asthma outcomes and reduced treatment satisfaction in prior research.^11^

Regarding potential determinants, we decided *a priori* to examine three basic demographic variables: age, sex and education. Education was measured by reports of the highest level of completed school education and was categorised into high (=general qualification for university entrance (‘Abitur’) or entrance qualification limited to universities of applied sciences (‘Fachhochschulreife’)), intermediate (=secondary school level I certificate (‘Mittlere Reife’)), low (=secondary modern school qualification (‘Haupt-/Volksschulabschluss), and no formal school degree. As earlier studies in patients with chronic disease had suggested that both the period of time since diagnosis and elevated anxiety/depression scores are associated with higher information needs,^12^ we additionally examined these two factors. The period of time since asthma diagnosis was operationalised on the basis of the reported year of diagnosis, which was subtracted from the year of survey participation. Symptoms suggestive of depression and anxiety were assessed by the German version of the PHQ-4,^20^ which measures each condition by two items.

In statistical analyses, participants aged ⩽45 years (i.e., the median) were categorised as young. The distribution of the education variable was skewed towards higher levels. We therefore categorised school education in ‘high’ versus ‘below’ (medium/low/no degree). On the PHQ-4, scores of three points or above on the respective subscale are considered to reflect depression or anxiety.^25^ Owing to our limited sample size, we combined both conditions into a single variable (depression and/or anxiety versus neither). Attempting to balance concerns of using meaningful versus statistically efficient categorisations, we decided *a priori* to define a short duration of asthma as 5 years or less since the diagnosis. We used multivariate linear regression models to examine associations of the potential determinants (all included into the same model) with the need scores.

## Figures and Tables

**Table 1:** Characteristics of the study population (*n*=189)

*Age,* n *(%)*	
⩽45 years	87 (47.55)
>45 years	96 (52.46)
	
Female, *n* (%)	146 (77.66)
	
*School education,* n *(%)*	
Low/medium/none	95 (51.08)
High	91 (48.92)
	
*Anxiety and depression*^a^, n *(%)*	
Neither	147 (79.89)
Either	37 (20.11)
	
*Years since asthma diagnosis,* n *(%)*	
⩽5 years	55 (30.39)
>5 years	126 (69.61)
	
*Reported needs in asthma treatment*^b^, n *(%)*	
Information on how asthma drugs operate^c^	86 (46.24)
Information from physician about side effects^c^	102 (54.84)
Information about interactions of asthma drugs with other medication^c^^d^	103 (67.76)
Information about how to take asthma medications^e^	23 (12.50)
Information about whether asthma inhaler may be taken more often than prescribed^e^	54 (29.03)
Practice under professional guidance how to handle your inhaler^e^	17 (9.04)
Information about what to do during an asthma attack^f^	60 (32.09)
Family members/friends know how to help during an asthma attack^f^	78 (41.49)
Practice under professional guidance breathing techniques^f^	79 (42.47)
Would like physician to ask about individual asthma triggers during diagnosis^g^	58 (30.85)
Would like physician to consider personal circumstances to a larger extent in asthma treatment^g^	71 (37.97)
Would like physician to consider patient’s asthma knowledge to a larger extent^g^	54 (28.88)
Would like physician to make more time in case of special requests^g^	77 (40.96)

a Measured by the Patient Health Questionnaire (PHQ)-4.

b Measured by the Needs in Asthma Treatment (NEAT) questionnaire.

c Items comprising the NEAT subscale ‘drug effects’.

d The denominator for that particular item was *n*=152 as responses were only provided by participants who took medication both for asthma and other conditions.

e NEAT subscale ‘handling drugs’.

f NEAT subscale ‘exacerbations’.

g NEAT subscale ‘patient expertise’.

**Table 2:** Determinants of patients’ needs in asthma treatment (multivariable linear regression analyses, mutually adjusted, *n*=189)^a^

*Exposure*	*Total need score*			*Exacerbations*			*Patient expertise*			*Handling asthma drugs*			*Drug effects*		
	b	*95% CI*	β	b	*95% CI*	β	b	*95% CI*	β	b	*95% CI*	β	b	*95% CI*	β
Female sex^b^	−0.05	−0.14, 0.05	−0.07	0.00	−0.14, 0.14	0.00	0.00	−0.14, 0.14	0.00	−0.07	−0.17, 0.03	−0.11	−**0.27**	−**0.44**, −**0.01**	−**0.27**
Younger age^c^	**0.13**	**0.05**, **0.21**	**0.25**	**0.15**	**0.03**, **0.27**	**0.20**	**0.17**	**0.06**, **0.28**	**0.23**	**0.13**	**0.04**, **0.21**	**0.23**	−0.01	−0.15, 0.13	−0.01
High education^d^	−0.06	−0.14, 0.02	−0.11	−0.06	−0.17, 0.06	−0.08	−0.05	−0.16, 0.06	−0.07	−0.06	0.15, 0.02	−0.12	−0.02	−0.16, 0.11	−0.03
Anxiety/depression^e^	**0.14**	**0.04**, **0.24**	**0.20**	**0.16**	**0.02**, **0.30**	**0.17**	**0.20**	**0.06**, **0.33**	**0.21**	0.08	−0.01, 0.18	0.13	0.05	−0.10, 0.21	0.06
Recent asthma diagnosis^f^	**0.13**	**0.05**, **0.21**	**0.22**	**0.19**	**0.07**, **0.32**	**0.23**	0.08	−0.04, 0.20	0.10	**0.11**	**0.02**, **0.19**	**0.18**	0.13	0.00, 0.27	0.16

Significant associations (*P*<0.05) are bold.

Abbreviations: *b*, unstandardised regression coefficient; CI, confidence interval for *b*; *ß*, standardised regression coefficient.

a Sample was restricted to participants who reported that they had been diagnosed with asthma, but not with chronic obstructive pulmonary disease.

b Female (coded as 1) versus male (coded as 0).

c Aged ⩽45 years (coded as 1) versus >45 years (coded as 0).

d Highest school education (coded as 1) versus medium/low/none (coded as 0).

e Depression, anxiety or both (coded as 1) versus neither (coded as 0).

f ⩽5 years (coded as 1) versus >5 years (coded as 0).

## Notes

AS is Joint Editor-in-Chief of *npj Primary Care Respiratory Medicine*, but was not involved in the editorial review of, nor the decision to publish this article. The other authors declare no conflict of interest.
